# Cognitive function and cardiometabolic disease risk factors in rural South Africa: baseline evidence from the HAALSI study

**DOI:** 10.1186/s12889-019-7938-z

**Published:** 2019-11-27

**Authors:** Brian Houle, Thomas Gaziano, Meagan Farrell, F. Xavier Gómez-Olivé, Lindsay C. Kobayashi, Nigel J. Crowther, Alisha N. Wade, Livia Montana, Ryan G. Wagner, Lisa Berkman, Stephen M. Tollman

**Affiliations:** 1 School of Demography, The Australian National University, ACT, Canberra, Australia; 2 MRC/Wits Rural Public Health and Health Transitions Research Unit (Agincourt), School of Public Health, Faculty of Health Sciences, University of the Witwatersrand, Johannesburg, 2193 South Africa; 3 Institute of Behavioral Science, University of Colorado Boulder, Boulder, CO USA; 4 Division of Cardiovascular Medicine, Brigham and Women’s Hospital, Boston, MA USA; 5 Harvard Medical School, Harvard University, Boston, MA USA; 6 Harvard Center for Population and Development Studies, Harvard University, Cambridge, MA USA; 7 INDEPTH Network, East Legon, Accra, Ghana; 8 Department of Epidemiology, School of Public Health, University of Michigan, Ann Arbor, MI USA; 9 Department of Chemical Pathology, National Health Laboratory Service, University of the Witwatersrand Faculty of Health Sciences, Johannesburg, South Africa; 10 Centre for Global Health Research, Umeå University, Umeå, Sweden; 11 Department of Global Health and Population, Harvard T.H. Chan School of Public Health, Boston, MA USA; 12 Departments of Social and Behavioral Sciences and Epidemiology, Harvard T.H. Chan School of Public Health, Boston, MA USA

**Keywords:** Africa, Cognitive function, Cross-sectional studies, Epidemiology, Noncommunicable disease, Cardiometabolic disease

## Abstract

**Background:**

Evidence on cognitive function in older South Africans is limited, with few population-based studies. We aimed to estimate baseline associations between cognitive function and cardiometabolic disease risk factors in rural South Africa.

**Methods:**

We use baseline data from “Health and Aging in Africa: A Longitudinal Study of an INDEPTH Community in South Africa” (HAALSI), a population-based study of adults aged 40 and above in rural South Africa in 2015. Cognitive function was measured using measures of time orientation, immediate and delayed recall, and numeracy adapted from the Health and Retirement Study cognitive battery (overall total cognitive score range 0–26). We used multiple linear regression to estimate associations between cardiometabolic risk factors (including BMI, hypertension, dyslipidemia, diabetes, history of stroke, alcohol frequency, and smoking status) and the overall cognitive function score, adjusted for potential confounders.

**Results:**

In multivariable-adjusted analyses (*n* = 3018; male = 1520; female = 1498; median age 59 (interquartile range 50–67)), cardiometabolic risk factors associated with lower cognitive function scores included: diabetes (b = − 1.11 [95% confidence interval: − 2.01, − 0.20] for controlled diabetes vs. no diabetes); underweight BMI (b = − 0.87 [CI: − 1.48, − 0.26] vs. normal BMI); and current and past smoking history compared to never smokers. Factors associated with higher cognitive function scores included: obese BMI (b = 0.74 [CI: 0.39, 1.10] vs. normal BMI); and controlled hypertension (b = 0.53 [CI: 0.11, 0.96] vs. normotensive).

**Conclusions:**

We provide an important baseline from rural South Africa on the associations between cardiometabolic disease risk factors and cognitive function in an older, rural South African population using standardized clinical measurements and cut-offs and widely used cognitive assessments. Future studies are needed to clarify temporal associations as well as patterns between the onset and duration of cardiometabolic conditions and cognitive function. As the South African population ages, effective management of cardiometabolic risk factors may be key to lasting cognitive health.

## Background

In sub-Saharan Africa complex and evolving demographic, epidemiological, social and behavioral transitions are rapidly occurring [1, 2] and driving increases in non-communicable disease (NCD) risk factors, such as hypertension [3, 4]. NCDs cause the majority of global deaths, with the greatest burden concentrated in low and middle-income countries (LMICs) [5, 6]. NCDs are further predicted to increase in LMICs due to population aging and social and behavioral changes [6, 7].

Increasing evidence shows links between cardiometabolic disease risk factors, such as hypertension and diabetes, and subsequent cognitive decline among older adults [8–14]. For instance, hypertension is thought to increase risk of cognitive decline through factors such as disruption of vascular function and small-vessel disease [15]. Diabetes and obesity have also been associated with lower cognitive function through insulin resistance, inflammation, oxidative stress, and effects on vascular function [15–17]. Both NCD risk factors and cognitive disorders are major contributors to the global burden of disability [18]. However, there are limited studies in many LMICs, particularly in sub-Saharan Africa, on cognition and its relationship with NCD risk factors [19, 20].

While HIV/AIDS remains a major cause of premature mortality in sub-Saharan Africa, there have been recent mortality declines and rising life expectancy of adults due to the widespread rollout of antiretroviral therapy (ART) [1, 21–23]. The population of HIV-positive individuals is subsequently aging, with HIV prevalence increasing in older ages and decreasing in younger ones [24], along with possible changes in cardiovascular and other epidemiological risk profiles associated with ART [25]. Evidence from high-income countries suggests there are effects of ART on cardiometabolic disease risk factors [26, 27], which was largely borne out by results from a systematic review of studies on HIV and ART from sub-Saharan Africa [28]. For instance, ART use is known to alter body-fat distribution which may increase the risk of cardiometabolic diseases [29]. In addition, studies have shown that HIV infection is associated with endothelial dysfunction which may lead to atherosclerotic disease [30]. In summary, increases in life expectancy and population aging [31], coupled with high prevalence of cardiometabolic risk factors in older adults [32] highlight the need for greater evidence to identify and reduce the burden of disability associated with cognitive decline.

Evidence is increasingly needed from population-based studies around the world and especially from LMICs, including sub-Saharan Africa, in order to identify: the burden of cognitive decline, important and potentially modifiable risk factors, and target areas to reduce health burdens and inequities. The existing research on cognitive function in older Africans is limited with few population-based studies [19, 33–42]. In this study, we aim to describe the baseline associations between cardiometabolic disease risk factors and cognitive function from a population-based study of older adults in rural South Africa. In addition to examining associations of cognitive function with cardiometabolic risk factors, we adjust for a number of other related risks, including socio-demographic, behavioral and other factors.

## Methods

### Data and study population

We use data from “Health and Aging in Africa: A Longitudinal Study of an INDEPTH Community in South Africa” (HAALSI) [43]. HAALSI is a population-based study nested in the Agincourt Health and socio-Demographic Surveillance System (AHDSS) in rural Mpumalanga province in north eastern South Africa, adjacent to Mozambique [44]. The Medical Research Council/Wits University Rural Health and Health Transitions Research Unit (Agincourt) has been conducting annual census and vital events updates since 1992. In 2015 the study population was approximately 110,000 people residing in 32 villages. Although the area has experienced significant improvements in socioeconomic circumstances since democratic change in 1994 [45], the population still has limited access to infrastructure (such as tarred roads, potable water) and unemployment is high.

All men and women aged 40 years and older on July 1, 2014 who had been permanent residents in the Agincourt HDSS for at least 1 year (based on the 2013 census) were eligible. A total of 6281 individuals were randomly selected (stratified on sex). Of the sampled 6281 men and women, 5890 were identified as alive and residing in the Agincourt HDSS. A total of 5059 respondents completed the interview (response rate 85.9%). The relatively earlier cut-off at age 40 was chosen to be able to prospectively measure pre-disease pathways that emerge in mid-life and influence later health status, and the high prevalence of cardiometabolic risk factors in middle-aged adults in this setting [4].

### Measures

#### Cognitive function

Cognitive function was measured using brief cognitive screening tests adapted from the U.S. Health and Retirement Study (HRS) [36, 46]. The cognitive screening tests (along with all materials for the interview) were translated and back translated to the local Shangaan language [36]. They were also pilot tested for accuracy, comprehension, and appropriateness with a sample of older respondents from the HDSS who were not part of the final HAALSI sample [36]. There were a total of five tests: (1) time orientation: respondents were asked to report the current year, month, and day and the name of the current South African president (one point for each correct answer; four points total); (2) immediate word recall: respondents were asked to recall as many words as possible from 10 words read aloud by the interviewer (one point for each word correctly recalled; ten points total); (3) delayed word recall: respondents were asked to list the words recalled after about 1 min with an interceding question (one point for each word correctly recalled; ten points total); (4) counting: respondents were asked to count sequentially from one to 20 (one point); and (5) numerical patterns: respondents who successfully counted to 20 were asked to complete the numeric sequence starting with two, four, six (one point). The overall total score ranged from 0 to 26 points.

#### Health conditions

Detailed information on all health measurements has been described elsewhere [43]. In summary, total cholesterol, HDL, LDL, and glucose were measured using point of care instruments. Systolic and diastolic blood pressure were measured three times after the respondent had been seated for 5 min and with 2 min intervals between measurements. We used the mean of the second and third measurement to calculate systolic and diastolic blood pressure. HIV status was determined through HIV enzyme-linked immunosobent assays (ELISA) on dried blood spots and ART status on testing for traces of Lamivudine (3TC) and Emtricitabine (ETC) (threshold levels > 0.02 μg/ml). We also included respondent self-reported history of stroke.

Dyslipidemia was defined as: total cholesterol ≥6.21 mmol/L or HDL < 1.19 mmol/L or LDL > 4.1 mmol/L or if the respondent self-reported being on treatment. Body mass index (BMI; kg/m^2^) categories were defined as per standard definitions and based on measured height and weight: obese ≥30 BMI, overweight BMI 25 to < 30, normal BMI 18.5 to < 25, and underweight BMI < 18.5.

Treatment for hypertension and diabetes were based on respondent self-report. For hypertension we defined four categories based on having a mean systolic blood pressure of ≥140 mmHg or diastolic blood pressure of ≥90 mmHg and respondent’s self-reported treatment status: (1) Without hypertension, for respondents reporting not being on treatment and having blood pressure below the threshold; (2) hypertension, controlled on treatment, for respondents reporting being on treatment and having blood pressure below the threshold; (3) hypertension uncontrolled and not on treatment, for respondents reporting not being on treatment and having blood pressure above the threshold; and (4) hypertension, uncontrolled on treatment, for respondents reporting being on treatment and having blood pressure above the threshold. For diabetes we defined four categories based on having elevated random glucose ≥11.1 mmol/L and respondent’s self-reported treatment status: (1) without diabetes, for respondents reporting not being on treatment and having blood glucose below the threshold; (2) diabetes, controlled on treatment, for respondents reporting being on treatment and having blood glucose below the threshold; (3) diabetes uncontrolled and not on treatment, for respondents reporting not being on treatment and having blood glucose above the threshold; and (4) diabetes, uncontrolled on treatment, for respondents reporting being on treatment and having blood glucose above the threshold.

#### Sociodemographic factors

We included sex, age in 10-year categories, nationality (South African or Mozambican/other), marital status (never married, separated/divorced, widowed, or currently married) and employment status (employed, not working, or homemaker), education (no formal education, some primary, some secondary, or completed secondary or more), and quintiles of the household wealth and asset index. The wealth index has been used in a number of studies from the HDSS and was shown to perform similarly to other, more complicated asset-based indicators [45].

#### Behavioral and other risk factors

We used respondent self-reported alcohol frequency (categorised as does not currently drink, drinks less than daily, and drinks 5–6 days per week or daily) and smoking status (never smoked, former smoker, and current smoker). We used the Center for Epidemiological Studies Depression (CES-D) eight item scale for depressive symptoms (categorised as a score of 0, 1–2, and 3–8) [47].

### Analysis

We compared mean scores for the total cognitive score across age categories using ANOVA, and by sex and cardiometabolic risk factors using t-tests. We then estimated a multivariable-adjusted, complete case ordinary least squares regression on the total cognitive score. We estimated three sequential models (each adjusted for sex and age) to determine which factors were associated with the total cognitive score: (1) cardiometabolic risk factors, (2) sociodemographics, and (3) behavioral and other risk factors. To test the sensitivity of the results that account for co-morbid cardiometabolic risks, we also fit models including each cardiometabolic indicator separately (e.g., BMI categories only) adjusted for sex, age, sociodemographics, and behavioral and other risk factors. To examine potential age-dependent effects, we also fit an alternate set of models using the final, third model and testing interactions between age (categorized as < 55 and ≥ 55 years of age to ensure adequate cell sizes) and each cardiometabolic risk factor using likelihood ratio tests. We also tested for interactions between gender and each cardiometabolic risk factor using likelihood ratio tests. All analyses were completed in Stata 14 (StataCorp. 2015. *Stata Statistical Software: Release 14*. College Station, TX: StataCorp LP).

## Results

Table 1 shows socio-demographics, cardiometabolic risk factors, and behavioral and other risk factors for the 5059 study respondents. Obesity was more prevalent among women than men (41% vs. 16%). Hypertension (58% overall) and dyslipidemia (44% overall) prevalence was high for both sexes. Diabetes prevalence was approximately 11% overall and similar between men and women. Both currently smoking and consuming alcohol were more common among men than women.

**Table 1:** Socio-demographic characteristics, cardiometabolic and other risk factors characteristics, by sex, HAALSI participants, Agincourt, 2014–2015 (*n* = 5059)

	Male *n* = 2345		Female *n* = 2714		Total *n* = 5059	
	*N*	(%)	*N*	(%)	*N*	(%)
Age						
40–49	418	(17.8)	500	(18.4)	918	(18.1)
50–59	624	(26.6)	786	(29.0)	1410	(27.9)
60–69	643	(27.4)	661	(24.4)	1304	(25.8)
70–79	446	(19.0)	432	(15.9)	878	(17.4)
80+	214	(9.1)	335	(12.3)	549	(10.9)
Nationality						
South Africa	1663	(70.9)	1865	(68.8)	3528	(69.8)
Mozambique/other	682	(29.1)	844	(31.2)	1526	(30.2)
Missing	0		5		5	
Marital status						
Never married	166	(7.1)	124	(4.6)	290	(5.7)
Separated / divorced	300	(12.8)	350	(12.9)	650	(12.9)
Widowed	276	(11.8)	1264	(46.6)	1540	(30.5)
Currently married	1602	(68.3)	973	(35.9)	2575	(50.9)
Missing	1		3		4	
Employment status						
Employed (part or full time)	443	(18.9)	362	(13.4)	805	(16.0)
Not working	1709	(73.1)	2010	(74.3)	3719	(73.7)
Homemaker	186	(8.0)	335	(12.4)	521	(10.3)
Missing	7		7		14	
Education						
No formal education	957	(40.9)	1349	(49.9)	2306	(45.7)
Some primary	833	(35.6)	883	(32.7)	1716	(34.0)
Some secondary	314	(13.4)	260	(9.6)	574	(11.4)
Secondary or more	234	(10.0)	212	(7.8)	446	(8.8)
Missing	7		10		17	
Wealth asset index quintiles						
1st (lowest)	502	(21.4)	544	(20.0)	1046	(20.7)
2nd	455	(19.4)	546	(20.1)	1001	(19.8)
3rd	450	(19.2)	541	(19.9)	991	(19.6)
4th	457	(19.5)	550	(20.3)	1007	(19.9)
5th (highest)	481	(20.5)	533	(19.6)	1014	(20.0)
Obesity^a^						
No	1818	(84.2)	1487	(58.8)	3305	(70.5)
Yes	341	(15.8)	1043	(41.2)	1384	(29.5)
Missing	186		184		370	
Hypertension^b^						
No	1033	(45.5)	1019	(38.3)	2052	(41.6)
Yes	1239	(54.5)	1645	(61.7)	2884	(58.4)
Missing	73		50		123	
Dyslipidemia^c^						
No	1058	(55.1)	1332	(57.1)	2390	(56.2)
Yes	861	(44.9)	1000	(42.9)	1861	(43.8)
Missing	426		382		808	
Diabetes^d^						
No	1933	(90.3)	2220	(88.5)	4153	(89.4)
Yes	207	(9.7)	288	(11.5)	495	(10.6)
Missing	205		206		411	
HIV and ART status						
Negative	1614	(77.3)	1898	(77.2)	3512	(77.2)
Positive no ART	159	(7.6)	214	(8.7)	373	(8.2)
Positive on ART	316	(15.1)	346	(14.1)	662	(14.6)
Missing	256		256		512	
Alcohol frequency						
Does not currently drink	1431	(61.1)	2454	(90.5)	3885	(76.9)
Drinks less than daily	660	(28.2)	206	(7.6)	866	(17.1)
Drinks 5–6 days per week or daily	251	(10.7)	53	(2.0)	304	(6.0)
Missing	3		1		4	
Smoking status						
Never	1305	(55.7)	2668	(98.3)	3973	(78.6)
Former	586	(25.0)	35	(1.3)	621	(12.3)
Current	450	(19.2)	10	(0.4)	460	(9.1)
Missing	4		1		5	
CES-D score						
0	761	(33.4)	869	(32.8)	1630	(33.1)
1 to 2	1170	(51.3)	1292	(48.8)	2462	(49.9)
3 to 8	349	(15.3)	488	(18.4)	837	(17.0)
Missing	65		65		130	
Stroke						
No	2280	(97.3)	2627	(96.9)	4907	(97.1)
Yes	64	(2.7)	85	(3.1)	149	(2.9)
Missing	1		2		3	

^a^BMI ≥ 30

^b^SBP ≥ 140 mmHg or DBP ≥ 90 mmHG or self-reported on treatment

^c^Total cholesterol ≥ 6.21 mmol/L or HDL < 1.19 mmol/L or LDL > 4.1 mmol/L or self-reported on treatment

^d^Elevated random glucose ≥ 11.1 mmol/L or self-reported on treatment

Table 2 shows unadjusted mean comparisons by age, sex, and cardiometabolic risk factors for the total cognitive score. There were lower average cognitive scores among older respondents. Obese respondents and those with dyslipidaemia had higher total cognitive scores on average compared to respondents without those conditions. Respondents with hypertension and diabetes had poorer total cognitive scores on average compared to respondents without those conditions.

**Table 2:** Total cognitive function scores in the HAALSI cohort by sex, age, and cardiometabolic risk factors, HAALSI participants, Agincourt, 2014–2015

	Mean	SD	*p*-value
Overall	14.31	(4.06)	
Sex			0.062
Male	14.20	(4.15)	
Female	14.44	(3.96)	
Age			< 0.001
40–49	15.90	(3.83)	
50–59	14.98	(3.95)	
60–69	13.91	(3.71)	
70–79	12.81	(3.73)	
80+	10.71	(3.89)	
Obesity^a^			< 0.001
No	14.11	(4.06)	
Yes	14.92	(3.86)	
Hypertension^b^			0.023
No	14.47	(4.09)	
Yes	14.16	(4.01)	
Dyslipidemia^c^			0.010
No	14.07	(4.03)	
Yes	14.43	(4.03)	
Diabetes^d^			0.007
No	14.33	(4.05)	
Yes	13.75	(3.91)	

^a^BMI ≥ 30

^b^SBP ≥ 140 mmHg or DBP ≥ 90 mmHG or self-reported on treatment

^c^Total cholesterol ≥ 6.21 mmol/L or HDL < 1.19 mmol/L or LDL > 4.1 mmol/L or self-reported on treatment

^d^Elevated random glucose ≥ 11.1 mmol/L or self-reported on treatment

Table 3 presents the results for the three sequential linear regressions of the total cognitive score. Model 1 adjusted for sex and age and included all the cardiometabolic risk factors. Obese (b = 1.2 [95% CI 0.84, 1.57]) and overweight (b = 0.79 [CI 0.45, 1.14]) respondents had on average higher scores compared to normal BMI individuals. In contrast, underweight respondents had, on average, more than one-point lower scores compared to those with normal BMI (b = − 1.31 [CI -1.94, − 0.69]). There were no significant differences between those who were normotensive and controlled or uncontrolled hypertensives, irrespective of treatment status. Conversely, uncontrolled diabetics not on treatment had lower scores on average compared to non-diabetics (b = − 0.74 [CI -1.34, − 0.14]). Dyslipidemia was not associated with average cognitive scores (*p* = 0.56). Finally, respondents who reported having ever experienced a stroke had over a one point lower cognitive score compared to those who had not.

**Table 3:** Linear regression of cognitive function (total score) on: cardiometabolic indicators (M1); M1 + sociodemographic factors (M2); M2 + behavioral and other risk factors (M3), HAALSI participants, Agincourt, 2014–2015 (N = 3018). For each model (M1-M3), results presented are fully adjusted for all variables included in each model (including all the cardiometabolic indicators)

	M1: cardiometabolic indicators			M2: + sociodemographics			M3: + behavioral and other risk factors		
	Beta	95% CI	*p*-value	Beta	95% CI	*p*-value	Beta	95% CI	*p*-value
Cardiometabolic indicators									
BMI^a^									
Underweight	−1.31	[− 1.94, − 0.69]	< 0.001	− 1.08	[− 1.69, − 0.48]	< 0.001	− 0.87	[− 1.48, − 0.26]	0.005
Normal	Ref			Ref			Ref		
Overweight	0.79	[0.45, 1.14]	< 0.001	0.59	[0.25, 0.93]	0.001	0.56	[0.22, 0.90]	0.001
Obese	1.2	[0.84, 1.57]	< 0.001	0.82	[0.46, 1.17]	< 0.001	0.74	[0.39, 1.10]	< 0.001
Hypertension^b^									
Without hypertension	Ref			Ref			Ref		
Hypertension, controlled on Rx	0.4	[−0.04, 0.84]	0.072	0.48	[0.05, 0.90]	0.027	0.53	[0.11, 0.96]	0.013
Hypertension, uncontrolled not on Rx	−0.06	[− 0.38, 0.27]	0.737	− 0.08	[− 0.39, 0.24]	0.638	− 0.04	[− 0.36, 0.27]	0.79
Hypertension, uncontrolled on Rx	0.13	[−0.29, 0.55]	0.545	0.21	[−0.20, 0.62]	0.316	0.22	[−0.19, 0.62]	0.291
Dyslipidemia									
No	Ref			Ref			Ref		
Yes	0.08	[−0.19, 0.36]	0.564	0.07	[−0.20, 0.33]	0.628	0.06	[−0.20, 0.33]	0.649
Diabetes^c^									
Without diabetes	Ref			Ref			Ref		
Diabetes, controlled on Rx	−0.8	[−1.75, 0.15]	0.098	−1.08	[−2.00, −0.17]	0.021	−1.11	[−2.01, − 0.20]	0.017
Diabetes, uncontrolled not on Rx	−0.74	[−1.34, − 0.14]	0.015	− 0.69	[− 1.27, − 0.11]	0.02	−0.75	[− 1.33, − 0.18]	0.01
Diabetes, uncontrolled on Rx	− 0.22	[− 1.03, 0.59]	0.596	−0.3	[− 1.08, 0.48]	0.444	− 0.26	[− 1.03, 0.52]	0.513
Stroke									
No									
Yes	−1.19	[−2.04, −0.35]	0.006	− 1.16	[− 1.98, − 0.34]	0.005	−1.11	[− 1.92, − 0.30]	0.007
Cardiometabolic-related behavioral risk factors									
Alcohol frequency									
Does not currently drink							Ref		
Less than daily							−0.41	[−0.79, −0.03]	0.033
5–6 days per week/ daily							0.35	[−0.26, 0.96]	0.262
Smoking status									
Never							Ref.		
Former							−0.67	[−1.08, −0.25]	0.002
Current							−0.84	[−1.37, − 0.31]	0.002

M1: Adjusted for age and sex

M2: Adjusted for age, sex, nationality, marital status, employment status, education, wealth asset index

M3: Adjusted for age, sex, nationality, marital status, employment status, education, wealth asset index, HIV and ART status, and CES-D score

^a^obese ≥30 BMI; overweight BMI 25 to < 30; normal BMI 18.5 to < 25; underweight BMI < 18.5

^b^Without hypertension: having a mean systolic blood pressure of < 140 mmHg and diastolic blood pressure of < 90 mmHg and reported not on treatment; hypertension, controlled on Rx: having a mean systolic blood pressure of < 140 mmHg and diastolic blood pressure of < 90 mmHg and reported on treatment; Hypertension, uncontrolled not on Rx: having a mean systolic blood pressure of ≥140 mmHg or diastolic blood pressure of ≥90 mmHg and reported not on treatment; Hypertension, uncontrolled on Rx: having a mean systolic blood pressure of ≥140 mmHg or diastolic blood pressure of ≥90 mmHg and reported on treatment

^c^Without diabetes: having random glucose < 11.1 mmol/L and reported not on treatment; diabetes, controlled on Rx: having random glucose < 11.1 mmol/L and reported on treatment; diabetes, uncontrolled not on Rx: having elevated random glucose ≥11.1 mmol/L and reported not on treatment; diabetes, uncontrolled on Rx: having elevated random glucose ≥11.1 mmol/L and reported on treatment

We next included other sociodemographic factors in Model 2 (Table 3). After adjusting for sociodemographic factors, respondents with controlled hypertension averaged about 0.48 points higher on the cognitive score compared to those without hypertension (CI 0.05, 0.90). Both uncontrolled (b = − 0.69 [CI -1.27, − 0.11]) and controlled (b = − 1.08 [CI -2.00, − 0.17]) diabetics had lower scores on average compared to non-diabetics, whilst those with uncontrolled diabetes who reported being on treatment had similar scores (*p* = 0.44). While slightly attenuated, all of the previous associations between cardiometabolic indicators and cognitive function remained.

The third and final model included additional behavioral and other risk factors. The associations between the total cognitive score and cardiometabolic risk factors in Model 2 remained after adjusting for these other covariates. Never smokers had higher cognitive scores on average compared to former and current smokers.

Results in the models including each cardiometabolic indicator separately were similar to the final model adjusting for all cardiometabolic factors simultaneously (results not shown). Interactions between gender and BMI (*p* = 0.09), hypertension (*p* = 0.07), dyslipidemia (*p* = 0.14), and diabetes (*p* = 0.25) did not significantly improve model fit. Finally, in the alternate set of models testing differential effects by age, an interaction between hypertension and age significantly improved model fit (*p* < 0.001; see Additional file 1). Among those under 55 years of age, uncontrolled hypertensives on treatment averaged over one point higher on the cognitive score compared to those without hypertension (b = 1.13; *p* = 0.004). However, amongst those 55 years and older, there was no difference in cognitive score between uncontrolled hypertensives on treatment and those without hypertension (b = − 0.26; *p* = 0.30). Associations with the other hypertension groups were similar between each age group to those in the prior models. Interactions between age and cardiometabolic indicators of BMI (*p* = 0.11), dyslipidemia (*p* = 0.76), and diabetes (*p* = 0.75) did not significantly improve model fit.

## Discussion

Using data from a population-based study of adults aged 40 years and older in rural South Africa, we examined baseline associations between cardiometabolic disease risk factors and cognitive function. Cardiometabolic disease risk factors associated with lower cognitive function scores included having diabetes, being underweight, and current smoking and having a smoking history. Protective cardiometabolic factors included overweight and obesity, and having controlled hypertension. Associations with a history of stroke were similar to prior studies in this study context and other global settings.

Lower cognitive scores amongst underweight compared to normal weight individuals have also been shown in cross-sectional and longitudinal studies in sub-Saharan Africa [34, 38, 40, 42]. We found higher cognitive scores on average among overweight/obese individuals compared to those with normal BMI. Previous studies have also shown that obesity in later life is associated with improved cognition [48, 49], whilst in mid-life it is detrimental. Thus, a U.S. longitudinal study showed increased risk of dementia among those with high BMI at 40–45 years [50], and other studies have reported similar associations [51]. These studies highlight the importance of a life course approach to understanding how cardiometabolic risk factors may affect cognition, including the age when obesity is measured and the impact of aging on body composition [51] – which will become possible with HAALSI as new data collection waves are completed. A potential explanation for our finding of lower cognitive scores for underweight individuals may be that weight loss represents more of a consequence rather than a cause of cognitive decline, which has been suggested in previous studies [52, 53]. The relationship between BMI and cognitive function may therefore be nonlinear, where obesity in mid-life leads to later cognitive decline while changes associated with cognitive decline lead to declining BMI in older ages [15, 48]. The mechanisms through which obesity in mid-life may attenuate cognitive function involve possible changes in vascular function and increases in inflammation, insulin resistance, and oxidative stress [15–17]. Additionally, as obesity is a risk factor for increased mortality from cardiovascular disease and diabetes, higher obesity-related mortality may have resulted in a survivorship bias in the HAALSI cohort.

Reduced cognitive function amongst controlled and uncontrolled diabetics has also been shown in cross-sectional and longitudinal studies, largely in high-income countries [15, 54–58]. Evidence suggests little difference in preventing dementia or reducing cognitive decline by different treatment approaches [59, 60]. Evidence on the association between diabetes and cognitive decline in sub-Saharan Africa is scarcer. A survey of two cities in Central Africa found no association between dementia and diabetes in univariate analyses [34]. Further studies in the region are needed that assess the onset and duration of diabetes, and their relationship with treatment adherence, glycaemic control, and cognitive function.

Our findings of higher cognitive scores among controlled hypertensives may reflect differential healthcare access or utilization. This is also supported by the data showing higher cognitive scores among uncontrolled hypertensives on treatment in the younger age group, but not in the older age group. There is some evidence of prevention of cognitive decline with antihypertensive treatment [61–63]. The lack of difference in cognition between older uncontrolled hypertensives and those who are normotensive may reflect reverse causation. Thus, for younger adults, higher hypertension-related stroke mortality may have resulted in a survivorship bias among those observed in the HAALSI cohort [64]. Given the cross-sectional nature of our data we were also unable to examine the duration of high blood pressure among the respondents [15]. In general, longitudinal studies have shown associations between baseline blood pressure and resulting cognitive decline [9, 39].

The strengths of our study include making use of data from a large, population-based sample of rural South African adults ages 40 and older with a high response rate, and the inclusion of a range of factors potentially associated with cognitive function. As the HAALSI sample can be considered largely representative of older, black populations residing in rural areas of South Africa [65], our study represents key baseline associations with cardiometabolic risk factors using a harmonized cognitive assessment based on that used in the Health and Retirement Study [36, 46]. The assessments have been shown to be strongly predictive of dementia [66], and allow for comparisons across a diverse range of international partner studies [11]. The cognitive measures showed external validity in HAALSI, with expected associations with age and education [36], as well as psychometric validity [67]. This study is one of the first to provide evidence on these associations in a rural South African setting with a rapidly aging population. Our results provide a point of comparison with other studies conducted at different time periods and settings, and future longitudinal studies.

We also acknowledge several potential limitations. First, as an analysis of baseline cross-sectional data, we are unable to assess the temporality of the associations we observed; longitudinal data that will accumulate with subsequent HAALSI waves are needed to clarify the direction and timing of associations with cognitive function. There is also no widespread consensus on the most appropriate approach to measure cognitive function among older populations in sub-Saharan Africa. While our measures are validated and widely used and we adjusted for education and other sociodemographic factors that could differentially affect response to the measures, there could be residual differential cognitive test item functioning according to education, literacy, or related factors. To our knowledge these measures have not been applied in similar settings. Additionally, our brief cognitive screening tool focused on memory assessment, and does not capture aspects of cognitive function that have previously been shown to be especially sensitive to hypertension and other cardiovascular conditions, such as executive function [68, 69]. Future research utilizing a multi-domain cognitive assessment may detect stronger or different patterns of associations between cognitive performance and cardiovascular risk.

## Conclusions

Using a population-based study of older adults in rural South Africa, we showed associations between prevalent diabetes, underweight, and current smoking with lower cognitive function scores on average, making use of standardized clinical measurements and cut-offs and a widely used cognitive assessment. Our findings provide an important baseline for future longitudinal studies, and will allow comparisons with other study contexts, populations, and time periods in the sub-Saharan Africa region and beyond. There is limited information on the associations between cardiometabolic risk factors and cognitive function in sub-Saharan Africa. Given increasing social and demographic changes, a growing proportion of adults of older ages, and the aging of the HIV-positive population [24], longitudinal studies are needed to clarify temporal associations as well as patterns and trajectories between the onset and duration of cardiometabolic conditions and cognitive decline. This is essential to provide an evidence base for future health and social programs that respond to population aging and the accompanying challenges of cognitive decline.

## Supplementary information

**Additional file 1. **Linear regression of cognitive function (total score) on cardiometabolic indicators, sociodemographic factors, and behavioral and other risk factors, HAALSI participants, Agincourt, 2014–2015 (*N* = 3018). Model includes an interaction between hypertension and age group (< 55 years and ≥ 55 years of age).

## Data Availability

The datasets generated and/or analysed during the current study are available at the Harvard Center for Population and Development Studies (HCPDS) program website: www.haalsi.org.

## Abbreviations

ART: Antiretroviral therapy
BMI: Body mass index
CES-D: Center for Epidemiological Studies Depression
CI: Confidence interval
HAALSI: Health and Aging in Africa: A Longitudinal Study of an INDEPTH Community in South Africa
HDSS: Health and socio-demographic surveillance system
LMIC: Low and middle-income countries
NCD: Noncommunicable disease
