# Supplementary material for: Cognitive function and cardiometabolic disease risk factors in rural South Africa: baseline evidence from the HAALSI study
Source: BMC Public Health. 2019 Nov 27;19:1579. doi: 10.1186/s12889-019-7938-z (PMC6882146; doi:10.1186/s12889-019-7938-z)
Supplement: Supplementary file 1 — Additional file 1. Linear regression of cognitive function (total score) on cardiometabolic indicators, sociodemographic factors, and behavioral and other risk factors, HAALSI participants, Agincourt, 2014–2015 (N = 3018). Model includes an interaction between hypertension and age group (< 55 years and ≥ 55 years of age). [file 12889_2019_7938_MOESM1_ESM.pdf]

Additional file 1. Linear regression of cognitive function (total score) on cardiometabolic indicators, sociodemographic factors, and behavioral and other risk factors, HAALSI participants, Agincourt, 2014-2015 (N=3018). Model includes an interaction between hypertension and age group (<55 years and ≥55 years of age).

	Beta	95% CI	p-value
**Cardiometabolic indicators**			
BMI[a]			
Underweight	-0.91	[-1.52, -0.29]	0.004
Normal	Ref		
Overweight	0.69	[0.35, 1.03]	<0.001
Obese	0.91	[0.55, 1.27]	<0.001
Hypertension[b]			
Without hypertension	Ref		
Hypertension, controlled on Rx	0.61	[-0.26, 1.48]	0.171
Hypertension, not controlled not on Rx	-0.29	[-0.77, 0.19]	0.237
Hypertension, not controlled on Rx	1.13	[0.37, 1.90]	0.004
Hypertension[b] *X* age group ≥55 years of age			
Hypertension, controlled on Rx	-0.33	[-1.32, 0.65]	0.508
Hypertension, not controlled not on Rx	0.34	[-0.29, 0.97]	0.288
Hypertension, not controlled on Rx	-1.39	[-2.27, -0.51]	0.002
Dyslipidemia			
No	Ref		
Yes	0.1	[-0.17, 0.37]	0.47
Diabetes[c]			
Without diabetes	Ref		
Diabetes, controlled on Rx	-0.98	[-1.90, -0.06]	0.037
Diabetes, not controlled not on Rx	-0.79	[-1.38, -0.21]	0.007
Diabetes, not controlled on Rx	-0.14	[-0.93, 0.64]	0.717
Stroke			
No			
Yes	-1.02	[-1.84, -0.20]	0.015
**Cardiometabolic-related behavioral risk factors**			
Alcohol frequency			
Does not currently drink			
Less than daily	-0.47	[-0.85, -0.09]	0.016
5-6 days per week/ daily	0.42	[-0.20, 1.04]	0.18
Smoking status			
Never			
Former	-0.63	[-1.04, -0.21]	0.003
Current	-0.52	[-1.05, 0.01]	0.057

Adjusted for age, sex, nationality, marital status, employment status, education, wealth asset index, HIV and ART status, and CES-D score.

[a] obese ≥30 BMI; overweight BMI 25 to <30; normal BMI 18.5 to <25; underweight BMI <18.5.
[b] Without hypertension: having a mean systolic blood pressure of <140 mmHg and diastolic blood pressure of <90 mmHg and reported not on treatment; hypertension, controlled on Rx: having a mean systolic blood pressure of <140 mmHg and diastolic blood pressure of <90 mmHg and reported on treatment; Hypertension, not controlled not on Rx: having a mean systolic blood pressure of ≥140 mmHg or diastolic blood pressure of ≥90 mmHg and reported not on treatment; Hypertension, not controlled on Rx: having a mean systolic blood pressure of ≥140 mmHg or diastolic blood pressure of ≥90 mmHg and reported on treatment.
[c] Without diabetes: having random glucose <11.1 mmol/L and reported not on treatment; diabetes, controlled on Rx: having random glucose <11.1 mmol/L and reported on treatment; diabetes, not controlled not on Rx: having elevated random glucose ≥11.1 mmol/L and reported not on treatment; diabetes, not controlled on Rx: having elevated random glucose ≥11.1 mmol/L and reported on treatment.
